# Insights into the Structural Conformations of the Tau Protein in Different Aggregation Status

**DOI:** 10.3390/molecules28114544

**Published:** 2023-06-04

**Authors:** Luca Pinzi, Nicolò Bisi, Claudia Sorbi, Silvia Franchini, Nicolò Tonali, Giulio Rastelli

**Affiliations:** 1 Department of Life Sciences, University of Modena and Reggio Emilia, Via Giuseppe Campi 103, 41125 Modena, Italy; luca.pinzi@unimore.it (L.P.); claudia.sorbi@unimore.it (C.S.); silvia.franchini@unimore.it (S.F.); 2 Centre National de la Recherche Scientifique (CNRS), Université de Paris-Saclay, BioCIS, Bat. Henri Moissan, 17 Av. des Sciences, 91400 Orsay, France; bisinicolo@gmail.com (N.B.); nicolo.tonali@universite-paris-saclay.fr (N.T.)

**Keywords:** tau, protein conformations, tauopathies, Alzheimer’s disease

## Abstract

Tau is a protein characterized by large structural portions displaying extended conformational changes. Unfortunately, the accumulation of this protein into toxic aggregates in neuronal cells leads to a number of severe pathologies, collectively named tauopathies. In the last decade, significant research advancements were achieved, including a better understanding of Tau structures and their implication in different tauopathies. Interestingly, Tau is characterized by a high structural variability depending on the type of disease, the crystallization conditions, and the formation of pathologic aggregates obtained from in vitro versus ex vivo samples. In this review, we reported an up-to-date and comprehensive overview of Tau structures reported in the Protein Data Bank, with a special focus on discussing the connections between structural features, different tauopathies, different crystallization conditions, and the use of in vitro or ex vivo samples. The information reported in this article highlights very interesting links between all these aspects, which we believe may be of particular relevance for a more informed structure-based design of compounds able to modulate Tau aggregation.

## 1. Introduction

Tau is a highly soluble protein originating from the *MAPT* (microtubule-associated protein Tau) gene. This protein can be classified among the so-called “Intrinsically Disordered Proteins”, being characterized by large structural portions subjected to fast and extended conformational changes [1]. Owing to the presence of such disordered regions in the structure, Tau can: (i) participate to the modulation of the activity of various biological partners; (ii) play a key role in the stability of the cytoskeletal complex [1], and; (iii) be engaged in the regulation of several cellular signaling processes [2].

**Figure 1:**
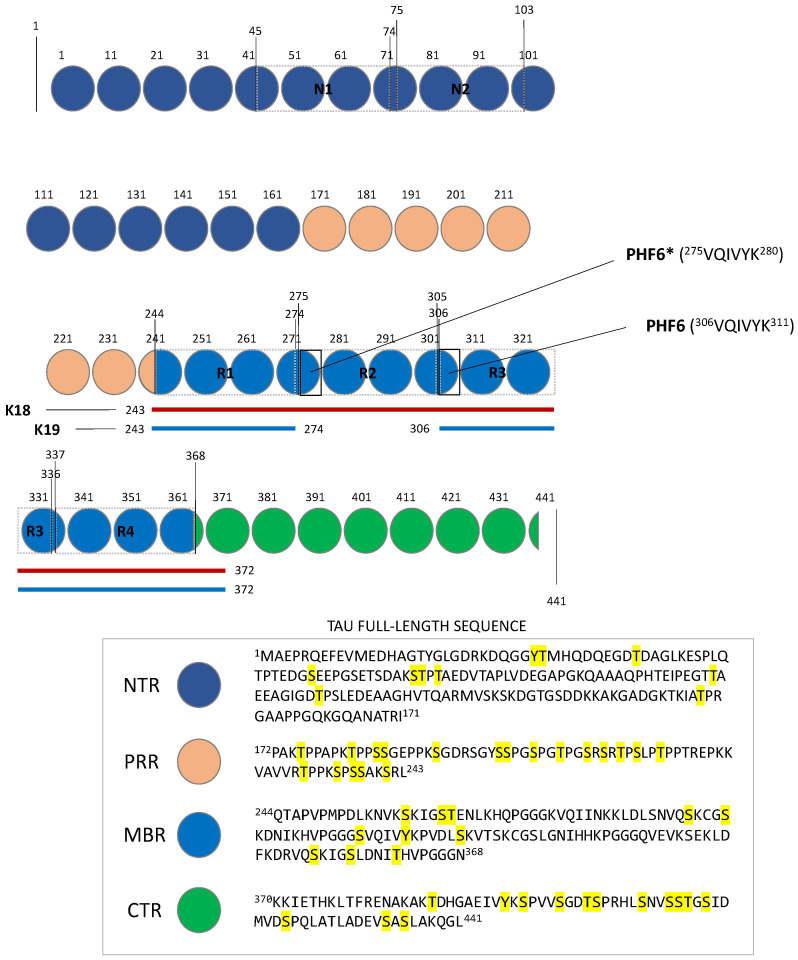
Schematic representation of the full-length Tau protein. N1 and N2, and R1–R4 represent the N-terminal and C-terminal inserts, respectively. According to the different RNA splicings, Tau can be expressed as 4R2N (Tau with N1, N2, R1, R2, R3, and R4 insertions), 4R1N (Tau with N1, R1, R2, R3, and R4 insertions), 4R0N (Tau with R1, R2, R3, and R4 insertions), 3R2N (Tau with N1, N2, R1, R3, and R4 insertions), 3R1N (Tau with N1, R1, R3, and R4 insertions), 3R0N (Tau with R1, R3, and R4 insertions). 0N, 1N and 2N isoforms represent around the 40%, 50%, and 10% of all Tau isoforms, respectively [23,24,25], PHF6 (VQIVYK) and PHF6* (VQIINK) are the regions of the protein involved in the formation of Tau aggregates [26]. K18 and K19 constructs of Tau including R1 to R4, R1, R3, and R4 insertions are also shown as red and blue bars, respectively. The full-length amino acids sequence of Tau, including phosphorylating sites (highlighted in yellow), is also reported [7,25].

At a structural level, four different regions were identified in the Tau structure, i.e., a N-Terminal Projection Region (NTR), a Microtubule-Binding Region (MBR) a proline-rich region (PRR), and Carboxyl-Terminal Region (CTR) (Figure 1), each of them being engaged in different biological functions. The CTR and MBR domains are highly conserved, and they contain similar structural motifs the length of 18 amino acids [3]; these regions constitute the main microtubule-interaction sites. The NTR and PRR regions are more disordered and less conserved, and they play a role in the modulation of a number of cellular functions including apoptosis [3]. The CTR region of Tau presents an amino acids sequence that is highly conserved across different species [4,5]. This portion, which was reported to be sensitive to phosphorylation (Figure 1) [3], facilitates the binding of Tau to microtubules and to heparin [3], and potentially participates to the regulation of microtubules dynamics [3]. The MBR region of Tau includes a high number of lysine residues, which makes it positively charged at physiological pH [5]. Such a portion of Tau plays a central role in the interaction with microtubules, which is likely to occur through the establishment of a number of electrostatic interactions between positively charged lysines and the negatively charged surface of microtubules [5,6]. Besides the binding to microtubules, MBR has also been reported to interact with a series of other proteins, including heat shock proteins and actin [3]. Of note, MBR is also sensitive to phosphorylation (e.g., serines 258, 262, 289, and 356 [7], Figure 1), albeit less than the nearby CTR and PRR regions, which, however, were reported to greatly affect the interaction of this protein with several binding partners [3,7,8]. The PRR region presents a high number of serine and threonine residues, similarly to the C-terminus portion of the protein [5]; it hosts several of the phosphorylation sites (Figure 1) affecting Tau binding and related physiology [3,7,8]. PRR is involved in the modulation of cell signaling, and axonal localization and phosphorylation [3]. The NTR region of Tau presents a negatively charged amino acids sequence due to the presence of a number of glutamate and aspartate amino acids. When Tau binds to the microtubules, this portion of the protein extends outside their surface and it interacts with other proteins, including annexins and 14-3-3 [3,9,10,11]. This portion of Tau binds to 14-3-3σ, heparin, and a number of other proteins in cells, and it was postulated to be involved in the modulation of cell signaling [3,12,13].

The more disordered regions of Tau include the first 200 and the last 80 amino acids of the N-terminal and C-terminal domains, respectively. These portions of the protein present different amino acids compositions and include a higher number of threonines and serines [3,14], which confer high structural flexibility [15]. The inherent flexibility of these portions hampered their study trough X-ray crystallography and Cryo-EM (Cryo-ElectroMicroscopy), or with other experimental techniques [16], as testified by the lack of high resolution three-dimensional (3D) structures of these regions of the protein into public repositories [17]. However, the more structured portions of Tau of relevance for its physiological role were identified [18].

To date, six and two major isoforms of Tau are currently known in the central and peripheral nervous systems, respectively [19,20], which vary in the number of N-terminal inserts (i.e., 0N, 1N, or 2N) in the NTR and C-terminal regions (i.e., 3R or 4R) at MBR (Figure 1). Additionally, two different constructs were also identified in the Tau structure, i.e., K18 and K19, which include residues Met243 to Glu372 (R1 to R4), and residues Met243 to Lys274 and Val337 to Glu372 (R1, R3 and R4), respectively (Figure 1). These truncated constructs of 4R and 3R Tau derive by a different splicing of exon 10 and were extensively studied in recent years, as they encompass the core of the paired helical filaments (PHFs) of this protein and are able to form amyloid fibrils [21,22].

Several studies demonstrated that different expression levels of Tau isoforms are present at different ages of brain development. Notably, the 0N3R Tau isoform is mainly expressed during neurogenesis, whereas adult brain presents nearly equal amount of all the six 3R and 4R isoforms [1,20,23], this balance being altered under pathological conditions [27]. According to recent findings, variations in the composition of the MBR region play a key role in the affinity of Tau for microtubules, the 4R isoform having the highest affinity [28]. The MBR domain contains also two hexapeptide motifs expressed in two different variants [29] at the second and third repeat insertions, namely PHF6 (^306^VQIVYK^311^) and PHF6* (^275^VQIINK^280^) [26], which act as nucleation centers of protein aggregation.

**Figure 2:**
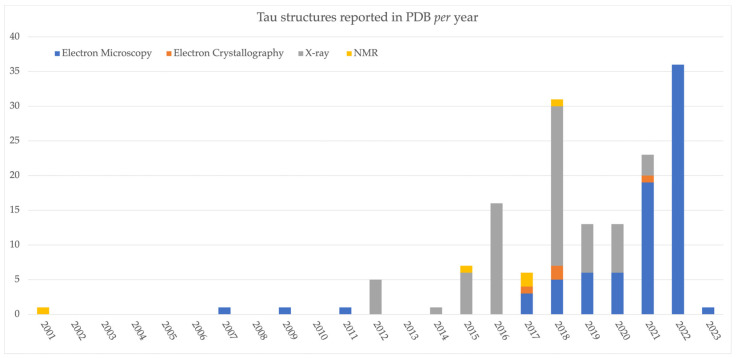
Three-dimensional (3D) complexes including Tau reported per year into the Protein Data Bank (PDB) [17]. As can be observed the majority of the reported complexes derive by X-ray crystallography and Electron microscopy experiments, the majority of Tau structures being obtained through this latter technique, in the last 4 years.

The possibility of Tau to adopt different conformations and to undergo post-translational modifications (the most frequently observed being phosphorylation, Figure 1) can lead to the formation of abnormal, toxic, aggregates in neuronal cells [30]. Research findings and clinical evidence showed that Tau aggregation follows a “prion-like” nucleation and elongation pathway, characterized by oligomers of different molecular weight and different phosphorylation levels [31]. Although Tau tends to fold differently in tauopathies, it is possible to identify common mechanisms and features in the formation of aggregates [32,33]. For example, tau phosphorylation levels are relatively reduced under physiological conditions, while studies performed on samples from patients with different tauopathies highlighted high degrees of Tau phosphorylation in the aggregates [34]. Moreover, when Tau dissociates from microtubules, it can dimerize through the formation of a series of interactions of PHF6 and PHF6* motifs, which eventually turn into oligomers. Subsequently, the oligomers collectively tend to form elongated structures known as protofilaments, which sort out in highly packed paired helical filaments, similar to those recently observed through Cryo-EM crystallography [35,36]. Such mechanism of aggregation can also be accelerated by biological events known as cross-seeding, where already formed amyloid structures facilitate the formation of other toxic aggregates [37]. Indeed, cross-seeding may explain some of events occurring during amyloids formation and it is under investigation in different Tau-related contexts. Several studies showed that Tau cross-seeding can occur in a homologous manner or heterologously; for example, with the Aβ peptide in AD [37,38]. The possibility to experimentally observe the conformations that Tau can adopt in pathological conditions is of central interest for the development of effective therapeutic treatments against different tauopathies. In this regard, significant efforts were devoted towards the determination of high-resolution structures of Tau, most of them being recently derived by electron microscopy experiments (Figure 2).

Additionally, the solved three-dimensional (3D) complexes of Tau provided useful insights into the conformations this protein can adopt in different aggregation status; it also clearly emerged how these structural data can be influenced by adopted experimental settings. On these premises, in this review, we first report an up-to-date classification of Tau structures according to their conformation and implication in different neurodegenerative diseases, according to the reported literature data. Moreover, we also provide insights into the different structural features of Tau from ex vivo and in vitro samples, discussing it with particular focus on structures related to Alzheimer’s disease (AD), which is one among the most widespread tauopathies affecting elderly people worldwide.

## 2. Tau Structures

**Figure 3:**
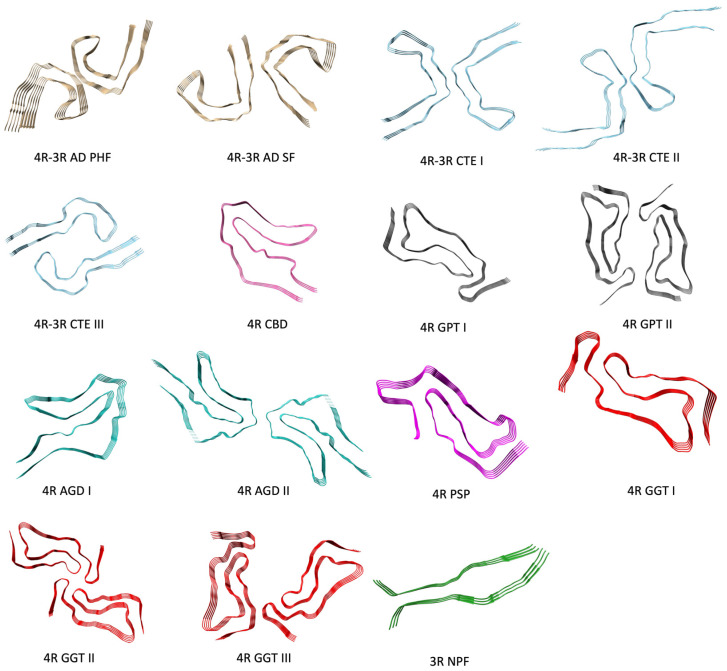
Representative conformations of Tau. In particular, the (4R–3R) AD PHF (PDB structure: 5O3L; N-Term: Val306—C-Term: Phe378), (4R–3R) AD SF (PDB structure: 5O3T; N-Term: Val306—C-Term: Phe378), (4R–3R) CTE I (PDB structure: 6NWP; N-Term: Ser305—C-Term: Arg379), (4R–3R) CTE II (PDB structure: 6NWQ; N-Term: Ser305—C-Term: Arg379), (4R–3R) CTE III (PDB structure: 7QL3; N-Term: Ile308—C-Term: Phe378), (4R) CDB (PDB structure: 6VHA; N-Term: Lys274—C-Term: Glu380), (4R) GPT I (PDB structure: 7P6A; N-Term: Gly272—C-Term: Arg379), (4R) GPT II (PDB structure: 7P6C; N-Term: Gly272—C-Term: Arg379), (4R) AGD I (PDB structure: 7P6D; N-Term: Gly273—C-Term: Asp387), (4R) AGD II (PDB structure: 7P6E; N-Term: Lys274—C-Term: Asn381), (4R) PSP (PDB structure: 7P65; N-Term: Gly272—C-Term: Asn381), (4R) GGT I (PDB structure: 7P66; N-Term: Gly272—C-Term: Arg379), (4R) GGT II (PDB structure: 7P67; N-Term: Gly272—C-Term: Arg379), (4R) GGT III (PDB structure: 7P68; N-Term: Gly272—C-Term: Arg379) and (3R) NPF (PDB structure: 6QJQ; N-Term: Lys274—C-Term: His330) are shown. N-terminal and C-terminal residues numbering refer to that of their respective crystal structures (see Table S1 in the Supporting Information for the complete list).

Recently, several structures of Tau were solved (Figure 3 and Table 1). In particular, they showed different assemblies characterizing known tauopathies, along with the different balance of 4R:3R isoforms. For example, 4R isoforms of Tau are predominant in the early stages of AD, i.e., before the oligomers progressively assemble into higher molecular weight and more organized PHF dimers.

These structures, together with the straight filaments (SF), form fibrils that are characteristic of late stage tauopathies, ultimately leading to AD and other neurodegenerative diseases [36,39].

**Table 1:** Structures of Tau reported in the Protein Data Bank. The structures are classified based on the presence of co-crystallized elements with Tau, and on the conformation and structural features of Tau in the complex. Authorships information related to the aligned Tau crystallographic complexes are also reported. Human crystal structures Tau (UniProt ID: P10636) were firstly downloaded from Protein Data Bank (https://www.rcsb.org/, accessed on 23 March 2023), leading to 131 entries. The collected entries were then split in their component chains, resulting in 158 Tau-single-chain complexes. Subsequently, the obtained structures were manually grouped according to their overall structural conformation. Afterwards, the entries in each group were aligned by using the “*Structure Alignment*” tool available in Maestro (Schrödinger suite 2018-3) with defaults settings [40]. Finally, data associated with the protein structures were also downloaded from PDB and processed to retain information on experimental conditions and authorship. The aligned complexes are provided as Supplementary Data.

Title	Type of Structure *	Class **	PDB Resolution (Å)	Publication Year	Experimental Method	References
7P6D	4R	AGD type 1	3.300	2021	Electron Microscopy	[41]
7P6E	4R	AGD type 2	3.400	2021	Electron Microscopy	[41]
6TJX	4R	CBD tau fibrils doublet	3.000	2020	Electron Microscopy	[42]
6VH7	4R	CBD tau fibrils doublet	3.800	2020	Electron Microscopy	[43]
6TJO	4R	CDB tau fibrils singlet	3.200	2020	Electron Microscopy	[42]
6VHA	4R	CDB tau fibrils singlet	4.300	2020	Electron Microscopy	[43]
6NWP	4R–3R	CTE I	2.300	2019	Electron Microscopy	[44]
8BYN	4R–3R	CTE I	2.600	2023	Electron Microscopy	[45]
7QL1	4R–3R	CTE I/II	3.340	2022	Electron Microscopy	[46]
6NWQ	4R–3R	CTE II	3.400	2019	Electron Microscopy	[44]
7QJW	4R–3R	CTE II	2.810	2022	Electron Microscopy	[46]
7QKX	4R–3R	CTE II	3.160	2022	Electron Microscopy	[46]
7QL0	4R–3R	CTE II	3.130	2022	Electron Microscopy	[46]
7QL3	4R–3R	CTE III	3.320	2022	Electron Microscopy	[46]
7QK5	4R (266/297–391)	CTE-like fold	1.920	2022	Electron Microscopy	[46]
7QKV	4R (266/297–391)	CTE-like fold	3.230	2022	Electron Microscopy	[46]
7P66	4R	GGT I	3.000	2021	Electron Microscopy	[41]
7P67	4R	GGT II	3.100	2021	Electron Microscopy	[41]
7P68	4R	GGT III	2.900	2021	Electron Microscopy	[41]
7QK3	4R (258–391)	GGT-like fold	2.440	2022	Electron Microscopy	[46]
7QK6	4R (258–391)	GGT-like fold	2.270	2022	Electron Microscopy	[46]
7QKG	4R (258–391)	GGT-like fold	3.360	2022	Electron Microscopy	[46]
7P6A	4R	GPT type 1a	1.900	2021	Electron Microscopy	[41]
7P6B	4R	GPT type 1b	2.200	2021	Electron Microscopy	[41]
7P6C	4R	GPT type 2	2.500	2021	Electron Microscopy	[41]
6QJQ	3R	Heparin-induced 3R	3.700	2019	Electron Microscopy	[47]
6QJP	4R	Heparin-induced 4R jagged	3.500	2019	Electron Microscopy	[47]
6QJH	4R	Heparin-induced 4R snake	3.300	2019	Electron Microscopy	[47]
6QJM	4R	Heparin-induced 4R twister	3.300	2019	Electron Microscopy	[47]
6CVJ	Tau fragment in complex with other proteins	Model of synthetic Tau	3.200	2018	Electron Microscopy	[6]
6CVN	Tau fragment in complex with other proteins	Model of synthetic Tau	3.900	2018	Electron Microscopy	[6]
7QJY	4R (266/297–391)	new I	3.140	2022	Electron Microscopy	[46]
7R4T	4R (266/297–391)	new I	2.750	2022	Electron Microscopy	[46]
7QJZ	4R (266/297–391)	new II	3.400	2022	Electron Microscopy	[46]
7QK2	4R (300–391)	new III	2.610	2022	Electron Microscopy	[46]
7QKZ	4R (305–379)	new III	2.650	2022	Electron Microscopy	[46]
7QKL	4R (266/297–391)	new IIX	2.070	2022	Electron Microscopy	[46]
7QKF	4R (266/297–391)	new IV	2.830	2022	Electron Microscopy	[46]
7QKM	4R (266–391, S356D)	new IX	2.660	2022	Electron Microscopy	[46]
7QKH	4R (258–391)	new V	3.170	2022	Electron Microscopy	[46]
7QKI	4R (297–408)	new VI	3.130	2022	Electron Microscopy	[46]
7QKJ	4R (266/297–391)	new VII	3.260	2022	Electron Microscopy	[46]
7QKU	4R (266/297–391)	new X	2.570	2022	Electron Microscopy	[46]
7QKW	4R (266–391, S356D)	new XI	2.320	2022	Electron Microscopy	[46]
7QKY	0N4R	new XII	1.860	2022	Electron Microscopy	[46]
7QL2	4R (266/297–391)	new XIII	2.950	2022	Electron Microscopy	[46]
7R5H	4R (266/297–391)	new XIV	2.590	2022	Electron Microscopy	[46]
7P65	4R	PSP	2.700	2021	Electron Microscopy	[41]
7U0Z	4R	PSP	4.200	2022	Electron Microscopy	[48]
7KQK (chains ABC)	Tau fragment in complex with other proteins	pTau fragment in complex with anti-pTau C21-ABS Fab	2.600	2020	X-ray Diffraction	
7KQK (chains HLP)	Tau fragment in complex with other proteins	pTau fragment in complex with anti-pTau C21-ABS Fab	2.600	2020	X-ray Diffraction	
2MZ7	Tau fragment in complex with other proteins	Tau bound to Microtubules		2015	Solution NMR (Model 1)	[49]
7PQC	Tau fragment in complex with other proteins	Tau bound to Microtubules	4.100	2021	Electron Microscopy	[50]
7PQP	Tau fragment in complex with other proteins	Tau bound to Microtubules	4.100	2021	Electron Microscopy	[50]
5O3L	4R–3R	Tau fibril from Alzheimer’s Disease—PHF	3.400	2017	Electron Microscopy	[35]
5O3O	4R–3R	Tau fibril from Alzheimer’s Disease—PHF	3.500	2017	Electron Microscopy	[35]
6HRE	4R–3R	Tau fibril from Alzheimer’s Disease—PHF	3.200	2018	Electron Microscopy	[51]
6VHL	4R–3R	Tau fibril from Alzheimer’s Disease—PHF	3.300	2020	Electron Microscopy	[43]
7MKF	4R–3R	Tau fibril from Alzheimer’s Disease—PHF	3.000	2021	Electron Microscopy	[52]
7MKH	4R–3R	Tau fibril from Alzheimer’s Disease—PHF	3.300	2021	Electron Microscopy	[52]
7NRQ	4R–3R	Tau fibril from Alzheimer’s Disease—PHF	2.760	2021	Electron Microscopy	[36]
7NRV	4R–3R	Tau fibril from Alzheimer’s Disease—PHF	3.000	2021	Electron Microscopy	[36]
7QJV	4R–3R	Tau fibril from Alzheimer’s Disease—PHF	3.290	2022	Electron Microscopy	[46]
7QJX	4R–3R	Tau fibril from Alzheimer’s Disease—PHF	2.990	2022	Electron Microscopy	[46]
7QK1	4R–3R	Tau fibril from Alzheimer’s Disease—PHF	3.030	2022	Electron Microscopy	[46]
7QKK	4R–3R	Tau fibril from Alzheimer’s Disease—PHF	2.800	2022	Electron Microscopy	[46]
7QL4	4R–3R	Tau fibril from Alzheimer’s Disease—PHF	3.200	2022	Electron Microscopy	[46]
7UPE	4R–3R	Tau fibril from Alzheimer’s Disease—PHF	3.400	2022	Electron Microscopy	[53]
7UPF	4R–3R	Tau fibril from Alzheimer’s Disease—PHF	3.300	2022	Electron Microscopy	[53]
7UPG	4R–3R	Tau fibril from Alzheimer’s Disease—PHF	3.800	2022	Electron Microscopy	[53]
7YMN	3R (266–391)	Tau fibril from Alzheimer’s Disease—PHF	3.460	2022	Electron Microscopy	[54]
5O3T	4R–3R	Tau fibril from Alzheimer’s Disease—SF	3.400	2017	Electron Microscopy	[35]
6HRF	4R–3R	Tau fibril from Alzheimer’s Disease—SF	3.300	2018	Electron Microscopy	[51]
6VI3	4R–3R	Tau fibril from Alzheimer’s Disease—SF	3.300	2020	Electron Microscopy	[43]
7MKG	4R–3R	Tau fibril from Alzheimer’s Disease—SF	3.070	2021	Electron Microscopy	[52]
7NRS	4R–3R	Tau fibril from Alzheimer’s Disease—SF	2.680	2021	Electron Microscopy	[36]
7NRT	4R–3R	Tau fibril from Alzheimer’s Disease—SF	2.680	2021	Electron Microscopy	[36]
7NRX	4R–3R	Tau fibril from Alzheimer’s Disease—SF	3.550	2021	Electron Microscopy	[36]
6GX5	3R	Tau fibril from PICK’s Disease—NPF	3.200	2018	Electron Microscopy	[55]
7YPG	3R	Spindle-like fibril	2.500	2022	Electron Microscopy	[54]
4FL5	Tau fragment in complex with other proteins	Tau fragment in complex with 14-3-3	1.900	2015	X-ray Diffraction	[12]
4Y32	Tau fragment in complex with other proteins	Tau fragment in complex with 14-3-3	1.700	2015	X-ray Diffraction	[56]
4Y3B	Tau fragment in complex with other proteins	Tau fragment in complex with 14-3-3	1.800	2015	X-ray Diffraction	[56]
4Y5I	Tau fragment in complex with other proteins	Tau fragment in complex with 14-3-3	1.400	2015	X-ray Diffraction	[56]
5BTV	Tau fragment in complex with other proteins	Tau fragment in complex with 14-3-3	1.700	2015	X-ray Diffraction	[12]
5HF3	Tau fragment in complex with other proteins	Tau fragment in complex with 14-3-3	1.800	2015	X-ray Diffraction	[56]
6FAU	Tau fragment in complex with other proteins	Tau fragment in complex with 14-3-3	1.250	2018	X-ray Diffraction	[57]
6FAV	Tau fragment in complex with other proteins	Tau fragment in complex with 14-3-3	1.400	2018	X-ray Diffraction	[57]
6FAW	Tau fragment in complex with other proteins	Tau fragment in complex with 14-3-3	1.400	2018	X-ray Diffraction	[57]
6FBW	Tau fragment in complex with other proteins	Tau fragment in complex with 14-3-3	1.450	2018	X-ray Diffraction	[57]
6FBY	Tau fragment in complex with other proteins	Tau fragment in complex with 14-3-3	1.500	2018	X-ray Diffraction	[57]
6FI4	Tau fragment in complex with other proteins	Tau fragment in complex with 14-3-3	2.000	2018	X-ray Diffraction	[57]
6FI5	Tau fragment in complex with other proteins	Tau fragment in complex with 14-3-3	1.700	2018	X-ray Diffraction	[57]
7EYC (chains ABQ)	Tau fragment in complex with other proteins	Tau fragment in complex with antigen	2.490	2021	X-ray Diffraction	
7EYC (chains LHP)	Tau fragment in complex with other proteins	Tau fragment in complex with antigen	2.490	2021	X-ray Diffraction	
5ZIA (chains ABC)	Tau fragment in complex with other proteins	Tau fragment in complex with CBTAU-24.1	2.600	2018	X-ray Diffraction	[58]
5ZIA (chains DEF)	Tau fragment in complex with other proteins	Tau fragment in complex with CBTAU-24.1	2.600	2018	X-ray Diffraction	[58]
5ZIA (chains GLR)	Tau fragment in complex with other proteins	Tau fragment in complex with CBTAU-24.1	2.600	2018	X-ray Diffraction	[58]
5ZIA (chains HIJ)	Tau fragment in complex with other proteins	Tau fragment in complex with CBTAU-24.1	2.600	2018	X-ray Diffraction	[58]
5ZIA (chains KMN)	Tau fragment in complex with other proteins	Tau fragment in complex with CBTAU-24.1	2.600	2018	X-ray Diffraction	[58]
5ZIA (chains OPQ)	Tau fragment in complex with other proteins	Tau fragment in complex with CBTAU-24.1	2.600	2018	X-ray Diffraction	[58]
5ZV3	Tau fragment in complex with other proteins	Tau fragment in complex with CBTAU-24.1	2.090	2018	X-ray Diffraction	[59]
6GK7	Tau fragment in complex with other proteins	Tau fragment in complex with CBTAU-27.1	2.950	2018	X-ray Diffraction	[59]
6GK8	Tau fragment in complex with other proteins	Tau fragment in complex with CBTAU-28.1	2.850	2018	X-ray Diffraction	[59]
5N5A	Tau fragment in complex with other proteins	Tau fragment in complex with F-actin		2017	Solution NMR (Model 4)	[60]
5N5B	Tau fragment in complex with other proteins	Tau fragment in complex with F-actin		2017	Solution NMR (Model 12)	[60]
5NVB	Tau fragment in complex with other proteins	Tau fragment in complex with F-actin		2018	Solution NMR (Model 1)	
4GLR (chains AHI)	Tau fragment in complex with other proteins	Tau fragment in complex with Fab	1.900	2012	X-ray Diffraction	[61]
4GLR (chains BJK)	Tau fragment in complex with other proteins	Tau fragment in complex with Fab	1.900	2012	X-ray Diffraction	[61]
4TQE	Tau fragment in complex with other proteins	Tau fragment in complex with Fab	1.600	2014	X-ray Diffraction	
5DMG (chains CDZ)	Tau fragment in complex with other proteins	Tau fragment in complex with Fab	2.500	2016	X-ray Diffraction	[62]
5DMG (chains EFX)	Tau fragment in complex with other proteins	Tau fragment in complex with Fab	2.500	2016	X-ray Diffraction	[62]
5DMG (chains HLP)	Tau fragment in complex with other proteins	Tau fragment in complex with Fab	2.500	2016	X-ray Diffraction	[62]
5E2V	Tau fragment in complex with other proteins	Tau fragment in complex with Fab	1.640	2016	X-ray Diffraction	[63]
5E2W	Tau fragment in complex with other proteins	Tau fragment in complex with Fab	1.500	2016	X-ray Diffraction	[63]
5MO3	Tau fragment in complex with other proteins	Tau fragment in complex with Fab	1.690	2016	X-ray Diffraction	
5MP1 (chains AHL)	Tau fragment in complex with other proteins	Tau fragment in complex with Fab	3.100	2016	X-ray Diffraction	
5MP1 (chains BCD)	Tau fragment in complex with other proteins	Tau fragment in complex with Fab	3.100	2016	X-ray Diffraction	
5MP1 (chains EFG)	Tau fragment in complex with other proteins	Tau fragment in complex with Fab	3.100	2016	X-ray Diffraction	
5MP1 (chains IJK)	Tau fragment in complex with other proteins	Tau fragment in complex with Fab	3.100	2016	X-ray Diffraction	
5MP3 (chains ABC)	Tau fragment in complex with other proteins	Tau fragment in complex with Fab	2.750	2016	X-ray Diffraction	
5MP3 (chains DHL)	Tau fragment in complex with other proteins	Tau fragment in complex with Fab	2.750	2016	X-ray Diffraction	
5MP5 (chains ABK)	Tau fragment in complex with other proteins	Tau fragment in complex with Fab	2.310	2016	X-ray Diffraction	
5MP5 (chains CD)	Tau fragment in complex with other proteins	Tau fragment in complex with Fab	2.310	2016	X-ray Diffraction	
5MP5 (chains EFI)	Tau fragment in complex with other proteins	Tau fragment in complex with Fab	2.310	2016	X-ray Diffraction	
5MP5 (chains HJL)	Tau fragment in complex with other proteins	Tau fragment in complex with Fab	2.310	2016	X-ray Diffraction	
6BB4 (chains HLP)	Tau fragment in complex with other proteins	Tau fragment in complex with Fab	2.100	2018	X-ray Diffraction	[64]
6BB4 (chains IMQ)	Tau fragment in complex with other proteins	Tau fragment in complex with Fab	2.100	2018	X-ray Diffraction	[64]
6BB4 (chains JNR)	Tau fragment in complex with other proteins	Tau fragment in complex with Fab	2.100	2018	X-ray Diffraction	[64]
6DC8	Tau fragment in complex with other proteins	Tau fragment in complex with Fab	1.800	2019	X-ray Diffraction	[65]
6DC9 (chains HLP)	Tau fragment in complex with other proteins	Tau fragment in complex with Fab	3.000	2019	X-ray Diffraction	[65]
6DC9 (chains IMQ)	Tau fragment in complex with other proteins	Tau fragment in complex with Fab	3.000	2019	X-ray Diffraction	[65]
6DCA (chains HLP)	Tau fragment in complex with other proteins	Tau fragment in complex with Fab	2.600	2019	X-ray Diffraction	[65]
6DCA (chains IMQ)	Tau fragment in complex with other proteins	Tau fragment in complex with Fab	2.600	2019	X-ray Diffraction	[65]
6DCA (chains JNR)	Tau fragment in complex with other proteins	Tau fragment in complex with Fab	2.600	2019	X-ray Diffraction	[65]
6LRA	Tau fragment in complex with other proteins	Tau fragment in complex with Fab	1.900	2020	X-ray Diffraction	[66]
6PXR	Tau fragment in complex with other proteins	Tau fragment in complex with Fab	1.560	2020	X-ray Diffraction	[67]
6XLI (chains ABE)	Tau fragment in complex with other proteins	Tau fragment in complex with Fab	2.000	2020	X-ray Diffraction	[68]
6XLI (chains CDF)	Tau fragment in complex with other proteins	Tau fragment in complex with Fab	2.000	2020	X-ray Diffraction	[68]
6XLI (chains HLP)	Tau fragment in complex with other proteins	Tau fragment in complex with Fab	2.000	2020	X-ray Diffraction	[68]
6H06 (chains ABK)	Tau fragment in complex with other proteins	Tau fragment in complex with FAB CBTAU-22.1	2.630	2018	X-ray Diffraction	[69]
6H06 (chains CDG)	Tau fragment in complex with other proteins	Tau fragment in complex with FAB CBTAU-22.1	2.630	2018	X-ray Diffraction	[69]
6H06 (chains EFJ)	Tau fragment in complex with other proteins	Tau fragment in complex with FAB CBTAU-22.1	2.630	2018	X-ray Diffraction	[69]
6H06 (chains HIL)	Tau fragment in complex with other proteins	Tau fragment in complex with FAB CBTAU-22.1	2.630	2018	X-ray Diffraction	[69]
1I8H	Tau fragment in complex with other proteins	Tau fragment in complex with Pin1 WW domain		2001	Solution NMR (Model 1)	[70]
7SP1	Tau fragment in complex with other proteins	Tau fragment in complex with RNA	3.400	2022	Electron Microscopy	[71]
5V5B	Tau fragment	Tau fragment KVQIINKKL	1.500	2018	Electron Crystallography	[72]
6NK4	Tau fragment	Tau fragment KVQIINKKL	1.990	2021	Electron Crystallography	[73]
6N4P	Tau fragment	Tau fragment RQEFEV	1.850	2021	X-ray Diffraction	[74]
6ODG	Tau fragment	Tau fragment SVQIVY	1.000	2019	X-ray Diffraction	[75]
4E0M	Tau fragment	Tau fragment SVQIVYK	1.750	2012	X-ray Diffraction	[76]
4E0N	Tau fragment	Tau fragment SVQIVYK	1.650	2012	X-ray Diffraction	[76]
4E0O	Tau fragment	Tau fragment SVQIVYK	1.820	2012	X-ray Diffraction	[76]
5V5C	Tau fragment	Tau fragment VQIINK	1.250	2018	Electron Crystallography	[72]
2ON9	Tau fragment	Tau fragment VQIVYK	1.510	2007	X-ray Diffraction	[77]
3OVL	Tau fragment	Tau fragment VQIVYK	1.810	2011	X-ray Diffraction	[78]
4NP8	Tau fragment	Tau fragment VQIVYK	1.510	2009	X-ray Diffraction	[79]
5K7N	Tau fragment	Tau fragment VQIVYK	1.100	2017	Electron Crystallography	[80]

Note: * residues composing the structures are reported within the round brackets. ** structures of Tau with conformations different to those observed in post-mortem samples are labelled as “new”.

To date, more than 130 structures of Tau are reported in the PDB. Several of them are in complexes of Tau with other proteins, including Fab and actin. The length of Tau filaments into such complexes ranges from 4 to 37 residues, with averages of 8 (“Tau Fragment” type of structures in Table 1) and 16 (“Tau fragment in complex with other proteins” type of structures in Table 1). Most of these complexes include PHF6 and PHF6*, and related phosphorylated sequences of Tau with selected antibodies, which are of great interest for the design of inhibitors of the aggregation of this protein. However, future efforts and experimental improvements in protein crystallography and cryo-EM spectroscopy will certainly help to provide also structures larger fragments of Tau. Additionally, structures including only Tau filaments present sequences of length ranging from 48 to 125 residues. Of note, several differences and similarities can be observed in these structures from a structural point of view (see below). The majority of Tau structures consist of one protomer, containing a variable number of equally stacked monomers of the protein, each of them with a sequence of the same length (e.g., PDB structure: 6VHA). However, structures including symmetrical and asymmetrical assemblies of two or more Tau protomers were also reported (e.g., PDB structures: 5O3O and 6QJQ); the formation of these assemblies mainly derives by the establishment of a series of hydrogen bonds and salt bridges interactions. An analysis of the secondary structure of these complexes revealed that none of them present amino acids in α-helices motifs, while all the Tau structures, and in particular the complexes classified as type “4R”, present higher percentages of amino acids in β-strands, followed by those labelled as “4R–3R” and “3R” (see Table 1 and Table S1 in the Supporting Information). Conversely, the percentages of amino acid residues in the sequences with a non-defined secondary structure were higher the complexes classified as “3R” (see Table 1 and Table S1 ) and lower in the “4R” type. Differences can also be observed in the percentages of residues of Tau performing hydrogen bond interactions in the complexes, which were, on average, significantly higher in the “4R” type of structures (see Table 1 and Table S1 ). Altogether, these data suggest that structures classified as type “4R” generally present a higher degree of structural compactness and definition, and a higher number of residues framed into defined networks of interactions, in line with data from the literature.

## 3. Tau Structures in Different Neurodegenerative Diseases

In pathological forms (e.g., when the protein presents mutations resulting in aberrant oligomerization), Tau ceases its usual protective function and ends up aggregating inside neurons to form large tangles that cause damage observed in the brain of affected patients [28]. Importantly, recent findings showed that Tau filaments from different individuals present the same structure, while different tauopathies tend to present different Tau folds [41,81,82]. On these premises, tauopathies can be classified via the isoforms that accumulate in Neurofibrillary tangles (NFTs) [41,81,82]. In this regard, Shy Y. et al. [41] very recently proposed a classification of tauopathies based on their folds complementing clinical diagnosis and neuropathology that allows the identification of novel types of aggregations, with respect to those previously known. Moreover, crystallographic data reported into the Protein Data Bank allowed to better highlight the key differences of Tau structures from the different pathologies (Table 1). In particular, except for frontotemporal dementias (FTDs) such as Pick’s disease (PiD), that mostly include aggregates of Tau 3R isoform, known tauopathies comprise all six 3R and 4R isoforms (e.g., Chronic Traumatic Encephalopathy—CTE—and Alzheimer’s disease—AD, Primary Age-Related Tauopathy—PART, Familial British Dementia—FDB, Familial Danish Dementia—FDD), or only Tau 4R isoforms (i.e., Corticobasal Degeneration—CBD, Progressive Supranuclear Palsy—PSP and Argyrophilic Grain Disease—AGD, Astrogliopathy—ARTAG, Globular Glial Tauopathy—GGT) [83,84,85,86].

The main factors driving the diverse folding of Tau and their role in the different tauopathies are still unclear. However, according to recently reported crystallographic data, similarities between different folds can be observed [41]. For example, Tau filaments from FDB and FDD and PART are structurally related to those observed from post-mortem samples of AD patients, while AGD filaments are similar to those of ARTAG [41]. Moreover, the filaments from globular glial tauopathy (GGT) are closely related to the structure of PSP, which is the second most common tauopathy after AD. Structures with features similar to GGT and PSP were also reported (i.e., GPT) [41]. Furthermore, Tau aggregates can also be classified as primary or secondary depending on whether they represent the main molecular lesion or are associated with other pathological features [87,88]. Aggregates formation depend on the presence of amyloid filaments in the human brain, patient population, age, and disease advancement. Therefore, it is becoming increasingly interesting to find a relationship between the various folds, type of neurodegenerative disease, and a clearer distinction between early and advanced disease phases [89].

The identification of protein structures forming amyloid filaments in the brain is of utmost importance for the identification of novel effective therapeutics against tauopathies. This is especially true considering that neurodegenerative disorders caused by aberrant aggregation of Tau affect millions of people around the world [33]. Cryo-EM spectroscopy was successfully employed to obtain high-resolution images of different forms of fibrils [35,36,41,46] from post-mortem brains of patients affected by nine different neurodegenerative diseases. Indeed, high-resolution structures of β-amyloid filament central to Alzheimer’s diseases are important to help dissecting events and find suitable drugs and biomarkers able to detect or modulate these phenomena [41,46]. For example, analysis of the assembly of Aβ peptides into filaments very recently revealed two S-shaped protofilaments formed in the human brain, each associated with either sporadic or familial Alzheimer’s disease [90]. Moreover, recent studies also helped to better understand how experimental approaches for the identification of Tau anti-aggregators adopted so far could provide potential artifacts or false positive readouts in bioassays and in protein crystallization [46,47]. This provided a significant step towards a better understating of how Tau aggregates in different disorders, and on the conditions behind the process of protein aggregation. For example, the crystal structures of Tau in complex with the fibril disaggregating agent epigallocatechin gallate (EGCG) (PDB structure: 7UPG) [53], as well as the PET ligand flortaucipir (PDB structure: 8BYN) [45] were recently reported. However, it should be noted that the interaction of these compounds with Tau was only partially solved. Further advances in experimental conditions and techniques will certainly help to fully disclose complexes of Tau with anti-aggregating agents at higher resolution, enabling more accurate structure-based design studies.

## 4. Comparison of the Structural Features of Tau from Ex Vivo and In Vitro Samples

With the advent of the Cryo-EM, which made it possible to analyze at atomic level the filaments of Tau extracted from the brain of AD patients (ex vivo samples), the comparison of their structure with the morphology of filaments, obtained during the in vitro aggregation in cell-free systems, became a practice to better understand if the process might reflect the same result in terms of morphology of the aggregates. In order to elucidate their similarities and differences, as well as to gain insights into the development of reliable in vitro models of tauopathies. In this section, we present a critical analysis of the structural features of Tau, starting from the PDB structures of filaments since 2017. In particular, we will focus on their morphology and structures, to dissect the principal differences and points in common between the ex vivo and in vitro material. Whenever possible, we will provide accurate details at the atomic level, in order to ease the recognition of the specific and important structural elements of these inclusions. This may help to evaluate their relevance in the existing models of tauopathies.

In 2017, Fitzpatrick et al. analyzed the intracellular neurofibrillary tangles from a cerebral cortex of an AD patient. The sarkosyl insoluble fraction presented various paired helical filaments (PHFs) and straight filaments (SFs), with a ratio of nearly 4:1. They were composed of full-length, hyperphosphorylated Tau and were morphologically characterized by immune-gold electron microscopy (EM) and cryo-EM (PDB structures 5O3L, 5O3O) [35]. PHFs showed a longitudinal spacing between crossovers of 650–800 Å and a width of 70–150 Å, while SFs presented a crossover distance between 700 and 900 Å with a width of 100 Å. Both ultrastructural polymorphs consist of two protofilaments, characterized by a common core, comprising a double helical stack of C-shaped subunits. The helical rise and the helical twist are 4.7 Å and −1°, respectively, for both filaments. The pronase-resistant core consists of residues V306–F378, which include R3 and R4 repeats, as well as 10 more residues in the C-terminal part, just after R4 residues (N368–F378). It is characterized by eight in-register parallel β-sheets, formed by a β-bend and a β-helix motif linked by β-strands. β1 is present at the very N-terminal, whose segment V306–K311 (PHF6) forms a packing interface with the complementary segment T373–F378 of β8. Following β1, β2 (V313–C322) is packed against β8 (N368-F378) through a zipper motif characterized by the presence of polar groups. β3 (N327–K331) is found in face of β7 (S356–V363), and its interaction is stabilized by hydrogen bonds between the sidechains of H328 and T361. Lastly, three β-strands present in R4, namely β4 (V337–S341), β5 (K343–K347), and β6 (R349–I354), are found in the β-bend and a β-helix motif imparting the previously identified C-shape motif [35]. The polymorphism of PHFs and SFs is due to differences in of the lateral surface contacts of the protofilaments. In PHFs, the two protofilaments form symmetric, identical structures. In particular, the interface is formed by the anti-parallel stacking of residues P332–Q336. Two hydrogen bonds between Q336 and K331 further stabilize the structure. Conversely, in the SFs, the protofilaments are organized in an a-symmetrical manner, with no H-bonds or salt bridges present at the interface [91].

One year later, Falcon et al. used Cryo-EM to image tau filaments extracted from the frontal cortex of several cases of sporadic, inherited, and atypical AD [51]. As in Fitzpatrick’s analysis, PHFs and SFs were present in a ratio of approximately 4:1 [35,51]. Regarding the core structure of the protofilaments, the same involvement of residues V306–F378 was observed, with the characteristic eight β-strands rich C-folding subunits, showing an insignificant variation in Tau filament structures between individuals with AD. The presence of this common core (V306–F378) was verified for all the samples analyzed by Cryo-EM deriving from three sporadic cases (irrespective of APOE genotype) and one inherited of AD. The higher resolution Cryo-EM structures of PHFs and SFs from case 2 showed the presence of two additional residues at the N-terminus, and two additional residues at the C-terminus of the ordered core of the protofilament. At the C-terminus, these residues were R379 and E380 from the sequence after R4. The interfaces between the two protofilaments of both PHFs and SFs were the same in all four cases. These findings show that the PHF and SF structures are identical between sporadic and inherited cases of AD [51].

In 2021, Shi et al. added the PET ligand APN-1607 to sarkosyl-insoluble tau filaments from the frontal cortex of an AD patient, in order to identify the binding hot-spot of the ligand [36]. This molecular complex was studied by Cryo-EM analysis. Again, authors found a heterogenous population of PHFs and SFs, with morphological and structural characteristics similar to the ones identified by Fitzpatrick et al. in 2017 (PDB structures: 7NRQ, 7NRS, 7NRT, 7NRV, and 7NRX) [35]. However, the binding of the ligand induced a conformational change allowing the C-shaped cavity expansion in SFs, compared to the PHF. Additionally, an approximate 12° difference in the turn between β6 and β7 was observed when comparing APN-1607 treated PHFs and SFs to the control samples (without APN-1607 ligation) [36].

**Figure 4:**
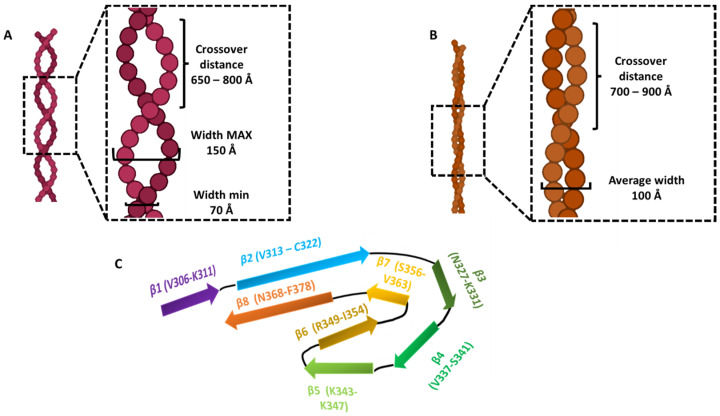
PHFs and SFs main morphological and structural characteristics from AD patients’ brain (ex vivo samples). Crossover distances and width filaments values are showed for PHFs (**A**) and SFs (**B**). A schematic representation of the protofilaments core, spanning residues V306–F378 and 8 8 β-sheets, is showed in (**C**).

So far, PHFs and SFs from ex vivo AD patients’ brain exhibited similar characteristics, independently from the research groups who investigated the samples and the individuals affected. These results confirmed the robustness of the Cryo-EM analysis and also the unique conformation of filaments extracted by patients bearing the same pathology and clinical symptoms. Therefore, this underlines a unique aggregation process leading to a particular morphology dependent only from the type of the disease where the aggregation is involved (Table 2). A visual representation pointing out the main features and differences between the two ultrastructural polymorphs, as well as their core structure, is shown in Figure 4.

The understanding of Tau aggregation, generally, comes mostly from in vitro studies using Tau models, in which exogenous additives are required to initiate the aggregation process. Authors tried several conditions for promoting Tau aggregation, and to develop models able to represent in a reliable way what may happen in cells and in vivo. The question if these filaments obtained in vitro are morphologically relevant with the disease arises spontaneously. We present here some of the recent highlights in this regard, with a particular focus on the morphology of the aggregates and their structural characterization.

As mentioned before, aggregation of full-length Tau requires exogenous promoters to take place. Generally, these agents share the common characteristics of being negatively charged. In fact, since Tau is positively charged at physiological pH, monomers tend to repel each other, thus preventing in vitro aggregation. By lowering Tau overall positive charge, polyanionic agents enhance protein–protein interaction, thus lowering the repulsion between monomers and easing the aggregation process. In this field, the most used co-factors are heparin, arachidonic acid, and RNA [92,93,94].

In 2022, Abskharon et al. induced the aggregation of full-length, wild type Tau using RNA as a promoter. The authors were able to solve the structure of fibrils with a resolution of 3.4 Å (PDB structure: 7SP1) by Cryo-EM [71]. Two polymorphs were identified: one twisted and one not twisted, which were present in a 4:1 ratio. Of the two, only the twisted polymorph was suitable for structure determination. The twisted tau-RNA fibrils were composed by two identical protofilaments and a cross-over distance of 829 Å. Also, they were characterized by a helical twist of 179.16° and a helical rise of 2.4 Å. Regarding their structure, filaments are composed by stacked in parallel, in-register β-sheets. The fibril core spans 36 residues nearby Tau C-terminal, namely E391–A426. In particular, the polymorph encompasses five β-strands. These are β1 (E391–V393), β2 (S396–S400), β3 (D402–Pro405), β4 (H407–S413), β5 (D418–V420). These residues are not involved in the core of AD patients’ fibrils previously found in ex vivo samples. In fact, the N-terminal and C-terminal domains of the protein are not folded in a well-defined secondary structure. Thus, they represent a flanking part protruding from fibrils, known as fuzzy coat. Concerning the surface interaction of the two components of the filament, β-sheet residues L408, N410, and S412 interact with identical residues of the opposing protofilament [71]. This peculiar polymorph may be interesting to further explore Tau aggregation and the dynamics behind it. In fact, since Tau–RNA interaction is relevant in cells and in vivo, the presence of this polymorph may act as a template to induce the formation of other Tau fibrils filaments. However, as it was not found in ex vivo samples, its implication in the Tau aggregation pathway is unclear and need to be further investigated.

In 2019, Zhang et al. analyzed by Cryo-EM different structures formed by the aggregation of full-length tau and its fragments in the presence of heparin [47]. Concerning Tau 2N3R, the construct corresponds to Tau full-length lacking the R2 repeat. It is one of the six isoforms of Tau present in the adult brain, and it is particularly relevant because it is found in tauopathies aggregates together with full-length Tau and other fragments [19,95]. Regarding filaments formed by full-length Tau, four different polymorphs were detected. The most representative was called “*snake*” and “*twister*”, and it was characterized by a crossover distance of 650 Å and a width between 40 and 100 Å. The second one displayed a crossover distance of 250 Å and width of 80 Å. The least common types bear the name of “*hose*” and “*jagged*”. Jagged filaments present a crossover distance of 450 Å, with width values comprised between 50 and 90 Å. Concerning hose filaments, morphological and structural features were not described.

Regarding filaments composed by Tau 2N3R, they were characterized by a crossover distance of 800 Å, and helical width values between 50 and 120 Å [47]. Concerning the different full-length Tau filaments structures at molecular level, the core of snake filament comprises residues G272–K330, which includes all R2 and R3 Tau repeats, forming six β-strands. Conversely, the core of twister filaments’ comprises residues K274–K321, consisting of R2 and half R3. Here, four β-strands are present. The same residues are found in the core of jagged filaments, but this time, it is composed of three β-strands. Contrary to the previous filaments, the Tau 2N3R polymorph does not present a defined symmetry between the two molecules that form the structure. One protofilament’s core is formed by K274–G330, while the other is composed by amino acids G272–G330, corresponding to R3 (in the 2N3R isoform, residues V275–S305 of R2 are not present). These residues form four β-strands. As we can see from these results, all full-length tau polymorphs share mostly the same core, with differences related to the β-strands content. In particular, they present the R2 region, which is not found in tau filaments of AD patients. The main different filament is the one represented by the 2N3R polymorph. Morphologically, its two protofilaments are not symmetrical. Moreover, because of the construct itself, it only presents R3 in the core. All in all, the Tau polymorphs formed in presence of heparin differ from the ex vivo filaments for different aspects. First, the core residues, which is composed of R3 and R4 in AD samples, and by R2 and R3 (or only R3 in case of Tau 2N3R) in the in vitro aggregates. Furthermore, parameters such as the cross-over distance, the width of the filaments and the helical twist, are also dissimilar. For example, the heparin-induced polymorphs present a shorter half pitch (crossover distance), and a thinner filament section in comparison with the ex vivo ones. This underlines the need of in vitro models that can more reliably match with tau in vivo aggregates. Taking this into account, an ideal cell-free model in absence of aggregation promoters should be developed in order to obtain filaments structurally similar to the ones found in AD patients’ brain. Indeed, without the use of aggregation cofactors, the morphology of the filaments will not be altered from exogenous influence. Moreover, by using constructs which lack regions not involved in the core structure (such as R2), it is possible to better understand the role of Tau repeats in the aggregation process and filament’s structure.

In 2022, Li et al. studied the aggregation of the Tau fragment 3R (Tau residues 266–391 without R2) and Tau 297–391 (also known as “dGAE model” [88]), without the presence of any aggregation promoter (PDB structures: 7YMN, 7YPG) [54]. The mixture of the two construct 3R and Tau 297–391 led to a majority of ribbon-like straight filaments. The other population was characterized by twisted fibrils. Among the latter, the less represented part displayed a half pitch of 79 nm and a width of 14 nm (“twisted type I” in Table 2). The remaining part exhibited a half pitch of 130 nm (“twisted type II” in Table 2). The aggregation of tau 297–391 (dGAE) also led to an important amount of ribbon-like straight fibrils. In the twisted population, 8% presented a half pitch of 76 nm and a width of 13 nm (“twisted type I” in Table 2). The other part showed half pitch values between 110 and 188 nm (twisted type II in Table 2). Under optimized aggregation condition of Tau 3R and Tau 297–391 together, authors were able to obtain PHF-like fibers as the major population. These filaments, whose shape is similar to the ones found in ex vivo samples, bear a half-pitch of 80 nm and a helical rise of 4.80 Å. When Tau 297–391 was used alone under the same aggregation conditions, a new PHF-like polymorph was discovered: this bears the name of “*spindle-like*” fibrils. The half pitch was found to be 75 nm, while the helical raise 4.82 Å. The filaments core is composed of residues G304–H362, which forms 7 β-strands, namely β1 (S305–K311), β2 (V313–K321), β3 (L325–H330), β4 (Q336–K340), β5 (E342–H346), β6 (R349–K353), β7 (L357–I359). Finally, the two protofilaments interface comprises residues C322–N358 [54].

These studies highlight how in vitro models were developed in order to understand the condition of in vivo Tau aggregation, and the morphology and structure of PHF and SF filaments. However, in current in vitro assays, Tau assembles into filaments intracellularly and requires the addition of anionic co-factors as sulphated glycosaminoglycans, RNA, fatty acids, and poly- glutamate, or heparin for facilitating its assembly into filaments [46,47]. Such additions can lead to the formation of polymorphic filaments, with structures different from those observed in the disease [46,47]. This leads to fibrils that are morphologically similar in terms of filament properties, but substantially different at the single molecule resolution. In fact, in patients AD samples, R3 and R4 tau repeats are normally found in the fibrils core, while in cell-free systems, R2 is usually comprised [47,71]. Recently, models based on Tau R3 and Tau dGAE (Tau 297–391) gained attention for the non-requirement of pro-aggregating molecules to form PHF-like filaments. Furthermore, aggregates shared quite the same morphological and structural features of ex vivo samples, making these models an attractive choice to study and understand Tau pathological aggregation in a cell-free environment. However, the shape of the filaments and their different polymorphic ratio do not always match those observed in AD patients’ materials [54].

## 5. Conclusions

The ability to obtain reliable, high-resolution Tau structures is of paramount importance for designing disease-modifying therapeutic agents with increased selectivity and sensitivity, as well as specific biomarkers for the different stages of the disease. In vitro assembly of Tau into disease-relevant filaments will facilitate studies to determine their roles in different neuropathologies. At the same time, the development of compounds that specifically bind to these structures or prevent their formation will be favored. Therefore, the molecular structures of Tau tangles are useful key factors to understand the various neurological diseases at a molecular level and will have large implications in diagnosis and treatment in the future, especially in the design of anti-aggregation ligands and biomarkers. To provide further insights into these aspects, in this review, we reported an up-to-date classification of Tau crystallographic complexes, relating them to different tauopathies. In particular, with regard to Alzheimer’s disease, we also discussed on the different features of the structures of this protein obtained from ex vivo and in vitro samples. Taken together, the information reported in this review article highlight very important links between available Tau structures, their involvement in different diseases, and the implications arising from comparison of structural features derived from ex vivo and in vitro samples. We believe that such information may facilitate a better understanding of such interconnections, with the hope that a new generation of compounds able to modulate Tau aggregation may arise from more informed and tailored structure-based designs.

## Figures and Tables

**Table 2:** Morphological and structural parameters describing tau filaments polymorphs from ex vivo and in vitro samples.

Type of Filaments	Width	Crossover Distance	Helical Rise	Helical Twist	Core Residues	Tau Repeats	Secondary Structure Elements	Sample Type	References
PHF	70–150 Å	650–800 Å	4.7 Å	−1°	V306–F378	R3, R4	8 β-sheets	Ex vivo AD patient, tau full-length	[35,36,55]
SF	100 Å	700–900 Å	4.7 Å	−1°	V306–F378	R3, R4	8 β-sheets	Ex vivo AD patient, tau full-length	[35,36,55]
Twisted	-	829 Å	179.16°	2.4°	E391-A426	C-terminal	5 β-sheets	In vitro (RNA induced aggregation), tau full-length	[71]
Not twisted	-	-	-	-	-	-	-	In vitro (RNA induced aggregation), tau full-length	[71]
Snake	40–100 Å	650 Å	-	−1.26°	G272–K330	R2, R3	6 β-strands	In vitro (heparin induced aggregation), tau full-length	[47]
Twister	80 Å	250 Å	-	−3.38°	K274–K321	R2, R3 (half)	4 β-strands	In vitro (heparin induced aggregation), tau full-length	[47]
Hose	-	-	-	-	-	-	-	In vitro (heparin induced aggregation), tau full-length	[47]
Jagged	50–90 Å	450 Å	-	−2.03°	K274–K321	R2, R3 (half)	3 β-strands	In vitro (heparin induced aggregation), tau full-length	[47]
2N3R tau filaments	50–120 Å	800 Å	-	−1.05°	K274–G330 and G272-G330 without V275–S305	R3	4 β-strands	In vitro (heparin induced aggregation), tau 2N3R	[47]
3R and tau 297–391 ribbon straight	-	-	-	-	-	-	-	In vitro 3R and tau 297–391	[54]
3R and tau 297–391 twisted type I	140 Å	790 Å	-	-	-	-	-	In vitro 3R and tau 297–391	[54]
3R and tau 297–391 twisted type II	-	1300 Å	-	-	-	-	-	In vitro 3R and tau 297–391	[54]
tau 297–391 ribbon straight	-	-	-	-	-	-	-	In vitro tau 297–391	[54]
tau 297–391 twisted type I	130 Å	760 Å	-	-	-	-	-	In vitro tau 297–391	[54]
tau 297–391 twisted type II	-	1100–1860 Å	-	-	-	-	-	In vitro tau 297–391	[54]
3R and tau 297–391 PHF-like	-	800 Å	4.80 Å	-	-	-	-	In vitro 3R and tau 297–391	[54]
tau 297–391 spindle-like	-	750 Å	4.82 Å	-	G304–H362	R3, R4	7 β-strands	In vitro tau 297–391	[54]

## Data Availability

Not applicable.

## Supplementary Materials

The following supporting information can be downloaded at: https://www.mdpi.com/article/10.3390/molecules28114544/s1. The aligned Tau crystallographic complexes are provided as Supplementary Data. Table S1: Structures of Tau reported within the Protein Data Bank. The structures are classified based on the presence of co-crystallized elements with Tau, and on the conformation and structural features of Tau in the complex. The percentages of bending residues, and of residues framed into alpha helices, beta strands and non-defined secondary structures are reported. Moreover, the percentage of Tau residues involved in hydrogen-bond interactions with other filaments of the same proteins in the complex are also reported. The percentages herein reported have been evaluated by means of an in house developed python script the BioPython (DOI: https: https://doi.org/10.1093/bioinformatics/btp163) and Pandas (DOI: https://doi.org/10.25080/Majora-92bf1922-00a) libraries. Numbering of the initial (“Start ResID”) and final (“Stop ResID”) residues of Tau sequences refers to those of their respective PDB complexes.
