# Supplementary material for: Insights into the Structural Conformations of the Tau Protein in Different Aggregation Status
Source: Molecules. 2023 Jun 4;28(11):4544. doi: 10.3390/molecules28114544 (PMC10254443; doi:10.3390/molecules28114544)
Supplement: Supplementary file 1 [file molecules-28-04544-s001.zip › molecules-2344251-supplementary.pdf]

SUPPORTING INFORMATION

Insights into the structural conformations of the tau protein in different aggregation status

Luca Pinzi,[1†] Nicolò Bisi,[2†] Claudia Sorbi,[1] Silvia Franchini,[1] Nicolò Tonali[2] and Giulio Rastelli[1,*]

[1] Department of Life Sciences, University of Modena and Reggio Emilia, Via Giuseppe Campi 103, 41125 Modena, Italy; giulio.rastelli@unimore.it (G.R.); silvia.franchini@unimore.it (S.F.); claudia.sorbi@unimore.it (C.S.); luca.pinzi@unimore.it (L.P.).

[2] Université de Paris-Saclay, CNRS, BioCIS, Bat. Henri Moissan17, av. des Sciences, 91400, Orsay, France; nicolo.tonali@universite-paris-saclay.fr (N.T.); bisinicolo@gmail.com (N.B.).

† Equally contributing authors

* Correspondence: giulio.rastelli@unimore.it; Tel.: +39-059-2058564

Table of Contents

Tables

Table S1 S3

Table S1: Structures of Tau reported within the Protein Data Bank. The structures are classified based on the presence of co-crystallized elements with Tau, and on the conformation and structural features of Tau in the complex. The percentages of bending residues, and of residues framed into alpha helices, beta strands and non-defined secondary structures are reported. Moreover, the percentage of Tau residues involved in hydrogen-bond interactions with other filaments of the same proteins in the complex are also reported. The percentages herein reported have been evaluated by means of an in house developed python script the BioPython (DOI: 10.1093/bioinformatics/btp163) and Pandas (DOI: 10.25080/Majora-92bf1922-00a) libraries. Numbering of the initial ("Start ResID") and final ("Stop ResID") residues of Tau sequences refers to those of their respective PDB complexes.

Title	*Type of Structure*	*Class*	Sequence length	% of bending residues	% of residues in alpha helics	% of residues in beta strands	% of residues in non-defined secondary structures	% of residues involved in H-bonds	Start ResID	Stop ResID
7P6D	4R	AGD type 1	115.0	8.7	0.0	53.0	38.3	75.1	GLY273	ASP387
7P6E	4R	AGD type 2	108.0	7.4	0.0	27.6	65.0	77.1	LYS274	ASN381
6TJX	4R	CBD tau fibrils doublet	107.0	11.2	0.0	40.2	48.6	0.0	LYS274	GLU380
6VH7	4R	CBD tau fibrils doublet	107.0	3.7	0.0	74.8	21.5	0.0	LYS274	GLU380
6TJO	4R	CDB tau fibrils singlet	107.0	7.5	0.0	15.9	76.6	0.0	LYS274	GLU380
6VHA	4R	CDB tau fibrils singlet	107.0	3.7	0.0	74.8	21.5	0.0	LYS274	GLU380
6NWP	4R – 3R	CTE I	75.0	6.9	0.0	66.0	27.1	74.7	SER305	ARG379
8BYN	4R – 3R	CTE I	75.0	9.3	0.0	61.3	29.4	75.1	SER305	ARG379
7QL1	4R – 3R	CTE I/II	73.0	11.0	0.0	47.9	41.1	76.7	VAL306	PHE378
6NWQ	4R – 3R	CTE II	75.0	9.3	0.0	27.6	63.1	31.3	SER305	ARG379
7QJW	4R – 3R	CTE II	75.0	8.0	0.0	58.7	33.3	79.5	SER305	ARG379
7QKX	4R – 3R	CTE II	73.0	10.1	0.0	61.6	28.3	76.5	VAL306	PHE378
7QL0	4R – 3R	CTE II	69.0	7.7	0.0	51.4	40.9	77.8	VAL309	THR377
7QL3	4R – 3R	CTE III	71.0	7.0	0.0	46.5	46.5	77.0	ILE308	PHE378
7QK5	4R (266/297–391)	CTE-like fold	74.0	7.2	0.0	64.9	27.9	76.7	VAL306	ARG379
7QKV	4R (266/297–391)	CTE-like fold	69.0	7.2	0.0	52.2	40.6	78.4	VAL309	THR377
7P66	4R	GGT I	108.0	7.4	0.0	55.2	37.4	75.8	GLY272	ARG379

7P67	4R	GGT II	108.0	7.4	0.0	56.3	36.3	83.5	GLY272	ARG379
7P68	4R	GGT III	108.0	6.5	0.0	56.0	37.5	79.8	GLY272	ARG379
7QK3	4R (258–391)	GGT-like fold	61.0	4.9	0.0	50.8	44.3	77.6	PRO270	HIS330
7QK6	4R (258–391)	GGT-like fold	54.0	3.7	0.0	55.0	41.3	79.6	VAL275	ILE328
7QKG	4R (258–391)	GGT-like fold	54.0	7.4	0.0	46.3	46.3	79.0	VAL275	ILE328
7P6A	4R	GPT type 1a	108.0	6.5	0.0	62.0	31.5	82.6	GLY272	ARG379
7P6B	4R	GPT type 1b	108.0	8.3	0.0	63.0	28.7	82.8	GLY272	ARG379
7P6C	4R	GPT type 2	108.0	8.3	0.0	61.1	30.6	84.0	GLY272	ARG379
6QJQ	3R	Heparin-induced 3R	58.0	0.0	0.0	26.8	73.3	0.0	GLY272	HIS330
6QJP	4R	Heparin-induced 4R jagged	48.0	0.0	0.0	52.1	47.9	0.0	LYS274	LYS321
6QJH	4R	Heparin-induced 4R snake	59.0	10.2	0.0	54.2	35.6	0.0	GLY272	HIS330
6QJM	4R	Heparin-induced 4R twister	48.0	0.0	0.0	45.8	54.2	0.0	LYS274	LYS321
6CVJ	Tau fragment in complex with other proteins	Model of synthetic Tau	12.0	8.3	0.0	0.0	91.7	66.7	VAL256	LYS267
6CVN	Tau fragment in complex with other proteins	Model of synthetic Tau	27.0	3.7	0.0	0.0	96.3	70.4	LYS274	VAL300
7QJY	4R (266/297–391)	new I	75.0	6.9	0.0	55.6	37.5	75.1	SER305	ARG379
7R4T	4R (266/297–391)	new I	76.0	10.9	0.0	61.2	27.9	75.4	GLY304	ARG379
7QJZ	4R (266/297–391)	new II	74.0	7.0	0.0	41.0	52.0	79.7	SER305	PHE378
7QK2	4R (300–391)	new III	71.0	11.5	0.0	60.6	27.9	75.1	ILE308	PHE378
7QKZ	4R (305–379)	new III	11.0	6.1	0.0	45.5	48.4	78.8	GLN307	LYS317
7QKL	4R (266/297–391)	new IIX	76.0	6.6	0.0	68.4	25.0	75.7	GLY304	ARG379
7QKF	4R (266/297–391)	new IV	41.5	7.4	0.0	53.9	38.7	73.5	VAL309	ILE360
7QKM	4R (266–391. S356D)	new IX	55.0	12.7	0.0	52.7	34.6	78.5	LYS274	ILE328
7QKH	4R (258–391)	new V	34.0	7.8	0.0	37.3	54.9	69.6	ILE277	TYR310
7QKI	4R (297–408)	new VI	76.0	7.0	0.0	52.9	40.1	76.1	GLY304	ARG379
7QKJ	4R (266/297–391)	new VII	75.0	10.7	0.0	49.8	39.5	80.5	SER305	ARG379
7QKU	4R (266/297–391)	new X	75.5	7.3	0.0	61.8	30.9	78.2	GLY304	ARG379
7QKW	4R (266–391. S356D)	new XI	51.0	8.7	0.0	55.7	35.7	77.4	VAL275	ILE328

7QKY	0N4R	new XII	66.0	15.2	0.0	59.1	25.7	77.6	LEU376	LEU441
7QL2	4R (266/297–391)	new XIII	50.0	4.0	0.0	68.7	27.3	74.7	VAL275	SER324
7R5H	4R (266/297–391)	new XIV	79.5	7.6	0.0	64.2	28.2	72.1	SER305	HIS388
7P65	4R	PSP	110.0	14.5	0.0	53.6	31.9	81.1	GLY272	ASN381
7U0Z	4R	PSP	110.0	11.8	0.0	50.9	37.3	66.1	GLY272	ASN381
7KQK (chains ABC)	Tau fragment in complex with other proteins	pTau fragment in complex with anti-pTau C21-ABS Fab	10.0	0.0	0.0	0.0	100.0	0.0	LYS224	PRO233
7KQK (chains HLP)	Tau fragment in complex with other proteins	pTau fragment in complex with anti-pTau C21-ABS Fab	10.0	0.0	0.0	0.0	100.0	0.0	LYS224	PRO233
2MZ7	Tau fragment in complex with other proteins	Tau bound to Microtubules	46.0	28.3	21.7	0.0	50.0	0.0	LYS267	PRO312
7PQC	Tau fragment in complex with other proteins	Tau bound to Microtubules	194.0	10.8	5.7	0.0	83.5	0.0	SER202	LYS395
7PQP	Tau fragment in complex with other proteins	Tau bound to Microtubules	194.0	13.4	6.2	0.0	80.4	0.0	SER202	LYS395
5O3L	4R – 3R	Tau fibril from Alzheimer's Disease - PHF	73.0	5.5	0.0	19.2	75.3	69.6	VAL306	PHE378
5O3O	4R – 3R	Tau fibril from Alzheimer's Disease - PHF	73.0	8.2	0.0	42.5	49.3	79.9	VAL306	PHE378
6HRE	4R – 3R	Tau fibril from Alzheimer's Disease - PHF	77.0	5.2	0.0	53.2	41.6	68.6	GLY304	GLU380
6VHL	4R – 3R	Tau fibril from Alzheimer's Disease - PHF	99.0	9.1	0.0	0.0	90.9	0.0	GLY304	GLY402
7MKF	4R – 3R	Tau fibril from Alzheimer's Disease - PHF	73.0	6.2	0.0	62.4	31.5	87.4	VAL306	PHE378
7MKH	4R – 3R	Tau fibril from Alzheimer's Disease - PHF	75.0	7.4	0.0	60.0	32.7	86.3	SER305	ARG379
7NRQ	4R – 3R	Tau fibril from Alzheimer's Disease - PHF	77.0	6.5	0.0	46.8	46.7	81.3	GLY304	GLU380
7NRV	4R – 3R	Tau fibril from Alzheimer's Disease - PHF	77.0	3.9	0.0	51.2	44.9	81.9	GLY304	GLU380
7QJV	4R – 3R	Tau fibril from Alzheimer's Disease - PHF	75.0	8.7	0.0	61.6	29.8	76.6	GLY304	PHE378
7QJX	4R – 3R	Tau fibril from Alzheimer's Disease - PHF	75.0	4.2	0.0	69.7	26.1	75.0	GLY304	PHE378
7QK1	4R – 3R	Tau fibril from Alzheimer's Disease - PHF	75.0	7.8	0.0	60.5	31.8	76.7	SER305	ARG379

7QKK	4R – 3R	Tau fibril from Alzheimer's Disease - PHF	76.0	6.6	0.0	47.8	45.6	70.6	GLY304	ARG379
7QL4	4R – 3R	Tau fibril from Alzheimer's Disease - PHF	76.0	7.9	0.0	63.2	28.9	76.8	GLY304	ARG379
7UPE	4R – 3R	Tau fibril from Alzheimer's Disease - PHF	74.0	5.4	0.0	71.6	23.0	81.5	VAL306	ARG379
7UPF	4R – 3R	Tau fibril from Alzheimer's Disease - PHF	74.0	5.4	0.0	71.6	23.0	81.5	VAL306	ARG379
7UPG	4R – 3R	Tau fibril from Alzheimer's Disease - PHF	75.0	5.3	0.0	62.7	32.0	81.2	SER305	ARG379
7YMN	3R (266-391)	Tau fibril from Alzheimer's Disease - PHF	76.0	5.3	0.0	13.2	81.5	61.2	GLY304	ARG379
5O3T	4R – 3R	Tau fibril from Alzheimer's Disease - SF	73.0	9.6	0.0	17.1	73.3	64.7	VAL306	PHE378
6HRF	4R – 3R	Tau fibril from Alzheimer's Disease - SF	77.0	9.1	0.0	42.9	48.0	62.5	GLY304	GLU380
6VI3	4R – 3R	Tau fibril from Alzheimer's Disease - SF	98.0	6.1	0.0	0.0	93.9	0.0	GLY304	GLY401
7MKG	4R – 3R	Tau fibril from Alzheimer's Disease - SF	73.0	6.2	0.0	64.4	29.5	84.5	VAL306	PHE378
7NRS	4R – 3R	Tau fibril from Alzheimer's Disease - SF	77.0	9.1	0.0	42.9	48.0	62.5	GLY304	GLU380
7NRT	4R – 3R	Tau fibril from Alzheimer's Disease - SF	77.0	3.9	0.0	57.8	38.4	80.0	GLY304	GLU380
7NRX	4R – 3R	Tau fibril from Alzheimer's Disease - SF	77.0	7.8	0.0	36.9	55.3	79.3	GLY304	GLU380
6GX5	3R	Tau fibril from PICK's Disease - NPF	125.0	8.0	0.0	48.8	43.2	55.7	LYS254	PHE378
7YPG	3R	Spindle-like fibril	59.0	5.1	0.0	27.1	67.8	66.1	GLY304	HIS362
4FL5	Tau fragment in complex with other proteins	Tau fragment in complex with 14-3-3	6.5	10.0	6.3	0.0	83.8	0.0	ARG211	PRO218
4Y32	Tau fragment in complex with other proteins	Tau fragment in complex with 14-3-3	6.5	0.0	0.0	0.0	100.0	0.0	ARG211	THR217
4Y3B	Tau fragment in complex with other proteins	Tau fragment in complex with 14-3-3	6.5	8.4	0.0	0.0	91.7	0.0	ARG211	THR217
4Y5I	Tau fragment in complex with other proteins	Tau fragment in complex with 14-3-3	6.5	8.4	0.0	0.0	91.7	0.0	ARG2	THR8
5BTV	Tau fragment in complex with other proteins	Tau fragment in complex with 14-3-3	4.0	0.0	0.0	0.0	100.0	0.0	GLY323	GLY326
5HF3	Tau fragment in complex with other proteins	Tau fragment in complex with 14-3-3	7.0	0.0	0.0	0.0	100.0	0.0	ARG2	THR8

6FAU	Tau fragment in complex with other proteins	Tau fragment in complex with 14-3-3	6.5	0.0	0.0	0.0	100.0	0.0	ARG2	GLY8
6FAV	Tau fragment in complex with other proteins	Tau fragment in complex with 14-3-3	6.5	0.0	0.0	0.0	100.0	0.0	ARG2	GLY8
6FAW	Tau fragment in complex with other proteins	Tau fragment in complex with 14-3-3	6.5	0.0	0.0	0.0	100.0	0.0	ARG2	GLY8
6FBW	Tau fragment in complex with other proteins	Tau fragment in complex with 14-3-3	6.5	0.0	0.0	0.0	100.0	0.0	ARG2	GLY8
6FBY	Tau fragment in complex with other proteins	Tau fragment in complex with 14-3-3	6.5	0.0	0.0	0.0	100.0	0.0	ARG2	GLY8
6FI4	Tau fragment in complex with other proteins	Tau fragment in complex with 14-3-3	4.0	0.0	0.0	0.0	100.0	0.0	PRO4	PRO7
6FI5	Tau fragment in complex with other proteins	Tau fragment in complex with 14-3-3	5.0	0.0	0.0	0.0	100.0	0.0	THR3	PRO7
7EYC (chains ABQ)	Tau fragment in complex with other proteins	Tau fragment in complex with antigen	8.0	0.0	0.0	0.0	100.0	0.0	ILE3	LEU10
7EYC (chains LHP)	Tau fragment in complex with other proteins	Tau fragment in complex with antigen	8.0	0.0	0.0	0.0	100.0	0.0	ILE3	LEU10
5ZIA (chains ABC)	Tau fragment in complex with other proteins	Tau fragment in complex with CBTAU-24.1	9.0	0.0	0.0	0.0	100.0	0.0	SER235	LEU243
5ZIA (chains DEF)	Tau fragment in complex with other proteins	Tau fragment in complex with CBTAU-24.1	9.0	0.0	0.0	0.0	100.0	0.0	SER235	LEU243
5ZIA (chains GLR)	Tau fragment in complex with other proteins	Tau fragment in complex with CBTAU-24.1	9.0	0.0	0.0	0.0	100.0	0.0	SER235	LEU243
5ZIA (chains HIJ)	Tau fragment in complex with other proteins	Tau fragment in complex with CBTAU-24.1	9.0	0.0	0.0	0.0	100.0	0.0	SER235	LEU243
5ZIA (chains KMN)	Tau fragment in complex with other proteins	Tau fragment in complex with CBTAU-24.1	9.0	0.0	0.0	0.0	100.0	0.0	SER235	LEU243
5ZIA (chains OPQ)	Tau fragment in complex with other proteins	Tau fragment in complex with CBTAU-24.1	9.0	0.0	0.0	0.0	100.0	0.0	SER235	LEU243
5ZV3	Tau fragment in complex with other proteins	Tau fragment in complex with CBTAU-24.1	12.0	0.0	25.0	0.0	75.0	0.0	GLU57	SER68
6GK7	Tau fragment in complex with other proteins	Tau fragment in complex with CBTAU-27.1	11.0	9.1	0.0	0.0	90.9	0.0	ILE308	VAL318
6GK8	Tau fragment in complex with other proteins	Tau fragment in complex with CBTAU-28.1	13.0	7.7	0.0	0.0	92.3	0.0	GLU57	THR69
5N5A	Tau fragment in complex with other proteins	Tau fragment in complex with F-actin	37.0	16.2	45.9	0.0	37.9	0.0	LYS254	LYS290

5N5B	Tau fragment in complex with other proteins	Tau fragment in complex with F-actin	28.0	21.4	17.9	0.0	60.7	0.0	GLY292	THR319
5NVB	Tau fragment in complex with other proteins	Tau fragment in complex with F-actin	15.0	13.3	26.7	0.0	60.0	0.0	LYS254	HIS268
4GLR (chains AHI)	Tau fragment in complex with other proteins	Tau fragment in complex with Fab	10.0	0.0	0.0	0.0	100.0	0.0	LYS225	LYS234
4GLR (chains BJK)	Tau fragment in complex with other proteins	Tau fragment in complex with Fab	10.0	0.0	0.0	0.0	100.0	0.0	LYS225	LYS234
4TQE	Tau fragment in complex with other proteins	Tau fragment in complex with Fab	16.0	12.5	0.0	0.0	87.5	0.0	LEU215	ARG230
5DMG (chains CDZ)	Tau fragment in complex with other proteins	Tau fragment in complex with Fab	9.0	0.0	0.0	0.0	100.0	0.0	VAL420	LEU428
5DMG (chains EFX)	Tau fragment in complex with other proteins	Tau fragment in complex with Fab	9.0	0.0	0.0	0.0	100.0	0.0	VAL420	LEU428
5DMG (chains HLP)	Tau fragment in complex with other proteins	Tau fragment in complex with Fab	9.0	0.0	0.0	0.0	100.0	0.0	VAL420	LEU428
5E2V	Tau fragment in complex with other proteins	Tau fragment in complex with Fab	9.0	22.2	0.0	0.0	77.8	0.0	GLY201	ARG209
5E2W	Tau fragment in complex with other proteins	Tau fragment in complex with Fab	7.0	14.3	0.0	0.0	85.7	0.0	PRO203	ARG209
5MO3	Tau fragment in complex with other proteins	Tau fragment in complex with Fab	8.0	25.0	0.0	0.0	75.0	0.0	LYS298	SER305
5MP1 (chains AHL)	Tau fragment in complex with other proteins	Tau fragment in complex with Fab	7.0	0.0	0.0	0.0	100.0	0.0	HIS299	SER305
5MP1 (chains BCD)	Tau fragment in complex with other proteins	Tau fragment in complex with Fab	7.0	0.0	0.0	0.0	100.0	0.0	HIS299	SER305
5MP1 (chains EFG)	Tau fragment in complex with other proteins	Tau fragment in complex with Fab	7.0	0.0	0.0	0.0	100.0	0.0	HIS299	SER305
5MP1 (chains IJK)	Tau fragment in complex with other proteins	Tau fragment in complex with Fab	7.0	0.0	0.0	0.0	100.0	0.0	HIS299	SER305
5MP3 (chains ABC)	Tau fragment in complex with other proteins	Tau fragment in complex with Fab	9.0	0.0	0.0	0.0	100.0	0.0	LYS298	VAL306
5MP3 (chains DHL)	Tau fragment in complex with other proteins	Tau fragment in complex with Fab	9.0	0.0	0.0	0.0	100.0	0.0	LYS298	VAL306
5MP5 (chains ABK)	Tau fragment in complex with other proteins	Tau fragment in complex with Fab	7.0	0.0	0.0	0.0	100.0	0.0	HIS299	SER305

5MP5 (chains CD)	Tau fragment in complex with other proteins	Tau fragment in complex with Fab	7.0	0.0	0.0	0.0	100.0	0.0	HIS299	SER305
5MP5 (chains EFI)	Tau fragment in complex with other proteins	Tau fragment in complex with Fab	7.0	0.0	0.0	0.0	100.0	0.0	HIS299	SER305
5MP5 (chains HJL)	Tau fragment in complex with other proteins	Tau fragment in complex with Fab	7.0	0.0	0.0	0.0	100.0	0.0	HIS299	SER305
6BB4 (chains HLP)	Tau fragment in complex with other proteins	Tau fragment in complex with Fab	7.0	0.0	0.0	0.0	100.0	0.0	ILE392	VAL398
6BB4 (chains IMQ)	Tau fragment in complex with other proteins	Tau fragment in complex with Fab	7.0	0.0	0.0	0.0	100.0	0.0	ILE392	VAL398
6BB4 (chains JNR)	Tau fragment in complex with other proteins	Tau fragment in complex with Fab	7.0	0.0	0.0	0.0	100.0	0.0	ILE392	VAL398
6DC8	Tau fragment in complex with other proteins	Tau fragment in complex with Fab	5.0	0.0	0.0	0.0	100.0	0.0	SER404	LEU408
6DC9 (chains HLP)	Tau fragment in complex with other proteins	Tau fragment in complex with Fab	6.0	0.0	0.0	0.0	100.0	0.0	THR403	LEU408
6DC9 (chains IMQ)	Tau fragment in complex with other proteins	Tau fragment in complex with Fab	6.0	0.0	0.0	0.0	100.0	0.0	THR403	LEU408
6DCA (chains HLP)	Tau fragment in complex with other proteins	Tau fragment in complex with Fab	6.0	0.0	0.0	0.0	100.0	0.0	THR403	LEU408
6DCA (chains IMQ)	Tau fragment in complex with other proteins	Tau fragment in complex with Fab	6.0	0.0	0.0	0.0	100.0	0.0	THR403	LEU408
6DCA (chains JNR)	Tau fragment in complex with other proteins	Tau fragment in complex with Fab	6.0	0.0	0.0	0.0	100.0	0.0	THR403	LEU408
6LRA	Tau fragment in complex with other proteins	Tau fragment in complex with Fab	6.0	0.0	0.0	0.0	100.0	0.0	VAL1	LYS6
6PXR	Tau fragment in complex with other proteins	Tau fragment in complex with Fab	8.0	0.0	0.0	0.0	100.0	0.0	ALA15	ASP22
6XLI (chains ABE)	Tau fragment in complex with other proteins	Tau fragment in complex with Fab	23.0	0.0	0.0	0.0	100.0	0.0	SER210	GLU222
6XLI (chains CDF)	Tau fragment in complex with other proteins	Tau fragment in complex with Fab	23.0	0.0	0.0	0.0	100.0	0.0	SER210	GLU222
6XLI (chains HLP)	Tau fragment in complex with other proteins	Tau fragment in complex with Fab	23.0	0.0	0.0	0.0	100.0	0.0	SER210	GLU222
6H06 (chains ABK)	Tau fragment in complex with other proteins	Tau fragment in complex with FAB CBTAU-22.1	7.0	0.0	0.0	0.0	100.0	0.0	VAL420	ALA426
6H06 (chains CDG)	Tau fragment in complex with other proteins	Tau fragment in complex with FAB CBTAU-22.1	7.0	0.0	0.0	0.0	100.0	0.0	VAL420	ALA426

6H06 (chains EFJ)	Tau fragment in complex with other proteins	Tau fragment in complex with FAB CBTAU-22.1	7.0	0.0	0.0	0.0	100.0	0.0	VAL420	ALA426
6H06 (chains HIL)	Tau fragment in complex with other proteins	Tau fragment in complex with FAB CBTAU-22.1	7.0	0.0	0.0	0.0	100.0	0.0	VAL420	ALA426
1I8H	Tau fragment in complex with other proteins	Tau fragment in complex with Pin1 WW domain	13.0	15.4	0.0	0.0	84.6	0.0	LYS1	SER13
7SP1	Tau fragment in complex with other proteins	Tau fragment in complex with RNA	36.0	8.3	0.0	0.0	38.9	80.9	GLU391	ALA426
5V5B	Tau fragment	Tau fragment KVQIINKKL	10.0	0.0	0.0	0.0	100.0	0.0	LYS274	ASP283
6NK4	Tau fragment	Tau fragment KVQIINKKL	9.0	0.0	0.0	0.0	100.0	22.2	LYS1	LEU9
6N4P	Tau fragment	Tau fragment RQEFEV	6.0	0.0	0.0	50.0	50.0	58.4	ARG1	VAL6
6ODG	Tau fragment	Tau fragment SVQIVY	6.0	0.0	0.0	0.0	100.0	0.0	SER1	TYR6
4E0M	Tau fragment	Tau fragment SVQIVYK	13.0	0.0	0.0	23.1	76.9	84.6	SER1	LYS13
4E0N	Tau fragment	Tau fragment SVQIVYK	13.0	0.0	0.0	23.1	76.9	84.6	SER1	LYS13
4E0O	Tau fragment	Tau fragment SVQIVYK	13.0	0.0	0.0	23.1	76.9	84.6	SER1	LYS13
5V5C	Tau fragment	Tau fragment VQIINK	6.0	0.0	0.0	0.0	100.0	0.0	VAL275	LYS280
2ON9	Tau fragment	Tau fragment VQIVYK	6.0	0.0	0.0	0.0	100.0	0.0	VAL1	LYS6
3OVL	Tau fragment	Tau fragment VQIVYK	6.0	0.0	0.0	0.0	100.0	16.7	VAL1	LYS6
4NP8	Tau fragment	Tau fragment VQIVYK	6.0	0.0	0.0	0.0	100.0	0.0	VAL1	LYS6
5K7N	Tau fragment	Tau fragment VQIVYK	6.0	0.0	0.0	0.0	100.0	0.0	VAL1	LYS6
